# XEN^®^-45 implantation for refractory uveitic glaucoma

**DOI:** 10.1007/s00417-023-06254-3

**Published:** 2023-10-19

**Authors:** Charlotte Evers, Alexandra Anton, Daniel Böhringer, Sara Kallee, Philip Keye, Thomas Neß, Heiko Philippin, Thomas Reinhard, Jan Lübke

**Affiliations:** 1 Eye Center, Medical Center – University of Freiburg, Faculty of Medicine, University of Freiburg, Killianstraße 5, 79106 Freiburg, Germany; 2 ADMEDICO Eye Center, Olten, Switzerland

**Keywords:** XEN^®^ implant, Glaucoma, Uveitis, Bleb revision

## Abstract

**Purpose:**

To evaluate the efficacy of XEN^®^-45 gel stent ab interno implantation for medically uncontrolled uveitic glaucoma.

**Methods:**

Retrospective analysis of 25 eyes receiving XEN^®^ gel stent for medically uncontrolled uveitic glaucoma from February 2019 to February 2023 with recording of intraocular pressure (IOP) values, ocular hypotensive medication, requirement for revision or secondary surgery and complications. Prerequisites for XEN^®^ implantation were a clear cornea, an open iridocorneal angle and an unscarred, mobile conjunctiva at the implantation site. Minimum follow-up required for inclusion was 3 months. The primary outcome measure was IOP compared to baseline. Complete and qualified success were defined as final IOP of ≤ 18 mmHg without or with topical antiglaucomatous treatment, respectively. Failure was defined as IOP > 18 mmHg on two consecutive visits, IOP reduction < 20%, persisting complications from hypotony and open conjunctival bleb revision. Further glaucoma surgical intervention was defined as complete failure.

**Results:**

Mean preoperative IOP was 35.3 ± 10.9 mmHg on 2.9 ± 0.9 topical antiglaucomatous agents. 19 of 25 patients (76%) received additional oral acetazolamide. 19 eyes were pseudophakic, 5 eyes phakic and 1 aphakic.

Early postoperatively, mean IOP reduced to 7.7 ± 3.0 mmHg (75.8% reduction). At final follow-up (mean 17.7 months) mean IOP was 12.0 ± 3.8 mmHg (62.5% reduction) on 0.2 ± 0.6 medications.

Six eyes (24%) required bleb revision at mean 28 weeks and therefore were categorized as failure. One eye failed despite bleb revision and restart of topical ocular hypotensive medication. Three other eyes (12%) had IOP spikes with uveitis flare-ups. Transient hypotony complications occurred in 32%. At final follow-up, 18 eyes (72%) achieved complete success and one eye (4%) qualified success.

**Conclusion:**

The XEN^®^ gel stent effectively reduced IOP in uncontrolled uveitic glaucoma, with 72% complete success. Bleb revision was required in 24%. IOP spikes occurred in 12% despite functioning blebs. Further follow-up is needed to determine long-term outcomes.


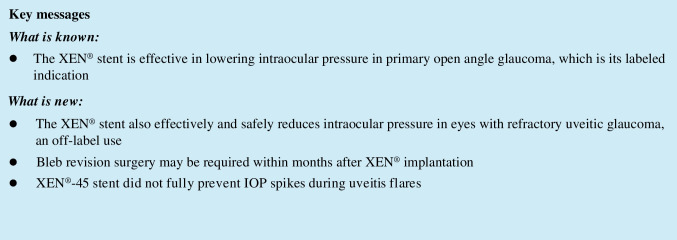


## Introduction

Glaucoma surgery in patients with uveitic glaucoma is particularly challenging. Pre-damaged tissue due to chronic/recurrent inflammation and possible perioperative uveitis flare-ups as well as high risk of scarring limit the chances of success.

To reduce the risk of intra- and postoperative complications, surgery should be minimally invasive, but still aim for a clear reduction of the mostly very high preoperative IOP. Compared to classical filtration surgery, the implantation of a XEN^®^-45 gel stent (Allergan, an Abbvie company, Irvine, CA, USA) is less invasive regarding iatrogenic trauma to iris, sclera and conjunctiva and may have a better safety profile.

The hollow cylindrical implant made of cross-linked collagen from porcine gelatin is injected ab interno via a clear corneal incision. The stent then drains fluid from the anterior chamber to the subconjunctival space. It has a length of 6 mm and a lumen of 45 µm. According to the Hagen-Poiseuille equation it has a pressure resistance of 6–8 mmHg [1].

XEN^®^-45 gel stent has been used successfully in primary open angle glaucoma [2–7], which it is labelled for. But there is limited data on its use in uveitic glaucoma (off-label use). Effectiveness for this indication has been shown in an exploratory prospective case series [8], as well as in urgent management [9].

We herein present our data of XEN^®^-45 gel stent implantation for uveitic glaucoma in a tertiary center in Germany.

## Methods

### Study patients

XEN^®^-45 gel stent (Allergan, an Abbvie company, Irvine, CA, USA) was implanted in 25 eyes of 24 patients with medically uncontrolled uveitic glaucoma by two surgeons (JL and CE). Patients were 17 to 79 years old (mean age: 55.4 years) at the time of XEN^®^ implantation. The prerequisites for XEN^®^ implantation were a clear cornea, an open iridocorneal angle and an unscarred, mobile conjunctiva at the implantation site. Although surgery was preferably performed in eyes with controlled inflammation for at least one month, some urgent cases were done without meeting this timeline. Minimum follow-up required for inclusion was 3 months.

### Surgical technique

The XEN^®^-45 gel stent was implanted ab interno via a clear corneal incision. Prior to implantation, a small amount of balanced salt solution, but no viscoelastic, was injected to widen the sub-Tenon’s space in the target quadrant. No blunt dissection of the conjunctiva was performed at the time of XEN^®^ implantation. After implantation and removal of intracameral viscoelastic, the anterior chamber was washed out with 1 ml of 4 mg/ml dexamethasone. We also injected 0.1 ml of 0.2 mg/ml mitomycin C subconjunctivally. If the subconjunctival portion of the XEN^®^ stent demonstrated restricted mobility, primary needling with a 27 gauge needle was performed. Postoperative treatment consisted of topical glucocorticoid drops (usually 1 mg/ml dexamethasone) 4–5 × daily and tapered based on the degree of postoperative conjunctival injection and intraocular inflammation.

Postoperative needling was undertaken if the XEN^®^ stent was encapsulated subconjunctivally but at the discretion of the surgeon could likely be released by needling alone. The criteria for bleb revision were signs of a dysfunctional bleb due to fibrotic tissue inhibiting the outflow through the subconjunctival portion of the XEN^®^ stent.

For open conjunctival bleb revision, a conjunctival peritomy was made at the limbus, followed by careful dissection of fibrotic tissue around the XEN^®^ stent. After verifying good flow through the XEN^®^ stent, it was placed underneath the Tenon’s fascia. Finally, the conjunctiva was closed with 7–0 Vicryl sutures, and 0.1 ml of dexamethasone 4 mg/ml was injected subconjunctivally.

After postoperative needling and bleb revision, patients received subconjunctival 5-fluorouracil injections (1 ml of 1% solution) for three consecutive days following surgery and again a course of glucocorticoid eye drops.

### Outcome measures, definition of successs and failure

The primary outcome measure was the final IOP and IOP reduction compared to baseline. Secondary outcome measures were the long-term requirement for IOP lowering medication, bleb revision surgery or secondary glaucoma surgery and complications.

Success was defined as a final IOP ≤ 18 mmHg and an IOP reduction ≥ 20% without (complete success) or with (qualified success) ocular hypotensive medications. Failure was defined as persistently elevated IOP > 18 mmHg, an IOP reduction < 20% compared to baseline, or the need for open conjunctival bleb revision. Needling was not considered evidence of failure in this analysis. Complete failure was defined as the need for further glaucoma surgical intervention.

### Statistical analysis

The success of XEN^®^ gel stent implantation was determined using descriptive statistics, including Kaplan–Meier estimations of event rates over time. The criteria for failure in the Kaplan–Meier analysis were revision surgery with conjunctival dissection or restart of topical ocular hypotensive treatment. Both eyes were included in the analysis for patients undergoing bilateral XEN^®^ implantation.

## Results

### Preoperative IOP and glaucoma treatment

Eighteen eyes (72%) had previous glaucoma surgery, 7 of these multiple surgeries, including Trabectome^®^ (*n* = 18), cyclophotocoagulation (*n* = 4), Baerveldt implantation (*n* = 2) and selective laser trabeculoplasty (*n* = 1) (Table 1). Nineteen eyes were pseudophakic, 5 eyes phakic and 1 eye aphakic. In the two eyes with previous Baerveldt implants, the drainage devices had been placed in the inferior nasal quadrant. We were careful to implant the XEN® stents in the superior nasal quadrant away from the area of tube-related scarring.

**Table 1:** Overview of all cases

Case	Type of uveitis	Previous glaucoma surgeries (number of previous glaucoma surgeries)		IOP at baseline (mmHg)	IOP after 6 months (mmHg)	IOP reduction after 6 months (%)	IOP after one year (mmHg)	IOP reduction after one year (%)	IOP at final follow-up (mmHg)	IOP reduction at final follow-up (%)	Follow-up period (months)	decimal visual acuity at baseline	decimal visual acuity at final follow-up
8	Herpetic anterior uveitis	Trabectome	(1)	28			13	53.6	14	50.0	28	1.0	1.0
2	Posner Schlossmann Syndrome	-	(0)	55	11	80.0			11	80.0	5	0.5	1.0
3	Fuchs Uveitis Syndrome	Trabectome	(1)	48	12	75.0	13	72.9	14	70.8	18	1.0	0.63
4	Idiopathic anterior uveitis	-	(0)	38	20	47.4	23	39.5	10	73.7	35	0.63	0.32
5	Fuchs Uveitis Syndrome	Trabectome, Baerveldt-implant	(2)	31	14	54.8	12	61.3	11	64.5	44	0.7	0.63
6	Posner Schlossmann Syndrome	-	(0)	38	30	21.1	15	60.5	17	55.3	34	1.0	1.0
7	Ocular Sarcoidosis	Trabectome	(1)	28			18	35.7	18	35.7	12	0.8	0.8
8	Idiopathic anterior uveitis	-	(0)	21	8	61.9	11	47.6	9	57.1	15	0.4	0.5
9	Juvenile idiopathic arthritis associated uveitis	Trabectome, 1 × Cyclophotocoagulation	(2)	26	8		8	69.2	10	61.5	32	0.1	0.1
10	Fuchs Uveitis Syndrome	Trabectome	(1)	60		69.2			16	73.3	4	0.4	0.4
11	Fuchs Uveitis Syndrome	Trabectome	(1)	38			40	-5.3	7	81.6	14	1.0	0.8
12	Fuchs Uveitis Syndrome	Trabectome, Selective Laser Trabeculoplasty	(2)	32					9	71.9	4	0.8	0.6
13	Herpetic anterior uveitis	Trabectome, Baerveldt-implant	(2)	20	16	20.0	24	-20.0	21	-5.0	18	0.1	0.32
14	Posner Schlossmann Syndrome	Trabectome	(1)	49					8	83.7	4	0.6	0.8
15	Fuchs Uveitis Syndrome	Trabectome, multiple (10x) Cyclophotocoagulation	(11)	24					5	79.2	30	0.005	0.005
16	Posner Schlossmann Syndrome	Trabectome	(1)	33			22	33.3	10	69.7	18	1.0	1.0
17	Herpetic anterior uveitis	Trabectome	(1)	46	10	78.3	11	76.1	13	71.7	19	0.25	0.63
18	Herpetic anterior uveitis^a^	Trabectome	(1)	42	11	73.8	9	78.6	10	76.2	22	0.8	0.8
19	Herpetic anterior uveitis^a^	-	(0)	33	12	63.6	9	72.7	11	66.7	21	1.0	0.8
20	Idiopathic anterior uveitis	-	(0)	24	6	75.0	9	62.5	11	54.2	21	0.63	0.63
21	Uveitis intermedia	Trabectome	(1)	24	12	50.0	12	50.0	11	54.2	20	0.8	0.4
22	Fuchs Uveitis Syndrome	Trabectome, 1 × Cyclophotocoagulation	(2)	30	18	40.0			17	43.3	10	0.4	0.4
23	Herpetic anterior uveitis	Trabectome, 2 × Cyclophotocoagulation	(3)	35	15	57.1			16	54.3	7	0.03	0.07
24	Tuberculosis associated Panuveitis	-	(0)	50					10	80.0	5	0.1	0.32
25	Herpetic anterior uveitis	Trabectome	(1)	30					12	60.0	3	0.2	0.4
mean			(1.4)	35.3	13.5	57.8	15.6	49.3	12.0	62.5	17.7	0.6	0.6
	a: Right and left eye of the same patient												

Mean preoperative IOP was 35.3 ± 10.9 mmHg (range 20–60 mmHg) despite maximum tolerable medication (Tables 1, 2, and 3). Preoperative medication consisted of 2.9 ± 0.9 topical ocular hypotensive agents and in 19 of 25 cases (76%) patients received adjunctive oral acetazolamide of variable dosage. The patient with a preoperative IOP of 20 mmHg had prior documented IOP of 30 mmHg on maximum tolerable topical treatment before additional acetazolamide lowered it to 20 mmHg. The patient with a preoperative IOP of 21 mmHg had advanced and progressive glaucoma and was on maximum tolerable IOP-lowering treatment. All other eyes had uncontrolled IOPs over 21 mmHg.

**Table 2:** Outcome measures and cumulative success rates at final follow-up

Case	IOP at baseline (mmHg)	IOP at final follow-up (mmHg)	IOP reduction at final follow-up (%)	bleb revision	secondary glaucoma surgery	final IOP ≤ 18 mmHg	final IOP ≤ 14 mmHg	final IOP ≥ 6 mmHg	IOP reduction ≥ 20%	complete success	qualitative success	failure
1	28	14	50.0	-	-	x	x	x	x	x	-	-
2	55	11	80.0	-	-	x	x	x	x	x	-	-
3	48	14	70.8	x	-	x	x	x	x	-	-	x
4	38	10	73.7	-	-	x	x	x	x	x	-	-
5	31	11	51.6	-	-	x	x	x	x	x	-	-
6	38	17	60.5	x	-	x	-	x	x	-	-	x
7	28	18	35.7	-	-	x	-	x	x	-	x	-
8	21	9	57.1	-	-	x	x	x	x	x	-	-
9	26	10	65.4	-	-	x	x	x	x	x	-	-
10	60	16	73.3	-	-	x	-	x	x	x	-	-
11	38	7	81.6	x	-	x	x	x	x	-	-	x
12	32	9	71.9	-	-	x	x	x	x	x	-	-
13	20	21	-5.0	x	-	-	-	x	-	-	-	x
14	49	8	83.7	-	-	x	x	x	x	x	-	-
15	24	5	54.2	-	-	x	x	-	x	x	-	-
16	33	10	69.7	x	-	x	x	x	x	-	-	x
17	46	13	71.7	-	-	x	x	x	x	x	-	-
18	33	10	73.8	-	-	x	x	x	x	x	-	-
19	24	1x	63.6	-	-	x	x	x	x	x	-	-
20	50	11	75.0	x	-	x	x	x	x	-	-	x
21	24	10	88.0	-	-	x	x	x	x	x	-	-
22	30	11	54.2	-	-	x	x	x	x	x	-	-
23	30	12	60.0	-	-	x	x	x	x	x	-	-
24	35	17	43.3	-	-	x	-	x	x	x	-	-
25	38	16	54.3	-	-	x	-	x	x	x	-	-
mean	35.3	12.0	62.5									
				6/25	0/25	24/25	19/25	24/25	24/25	18/25	1/25	6/25
				24%	0%	96%	19%	96%	96%	72%	4%	24%

**Table 3:** IOP values, percentual IOP reduction and number of IOP lowering agents

	mean ± SD	range
preoperative IOP	35.3 ± 10.9 mmHg	20–60 mmHg
early postoperative IOP	7.7 ± 3.0 mmHg	1.8–13 mmHg
early postoperative IOP reduction	77.7 ± 12.3%	48.5–93.0%
IOP at around 12 months	15.6 ± 8.3 mmHg	8–40 mmHg
IOP reduction at around 12 months	49.3 ± 28.1%	-20.0–78.6%
IOP at final follow-up	12.0 ± 3.8 mmHg	5–21 mmHg
IOP reduction at final follow-up	62.5 ± 18.9%	-5.0–84%
number of topical IOP lowering agents		
- preoperative	2.9 ± 0.9	1–4
- at final follow-up	0.2 ± 0.6	0–2

### Uveitis types and treatment

The uveitis types included Fuchs uveitis syndrome (*n* = 7), herpetic anterior uveitis (*n* = 7), Posner-Schlossmann syndrome (*n* = 4), and others (*n* = 7) such as ocular sarcoidosis, juvenile idiopathic arthritis associated uveitis, intermediate uveitis, tuberculosis-associated uveitis and idiopathic chronic/recurrent anterior uveitis (Table 1).

At the time of surgery, 19 eyes (76%) were on one or more topical medications for their uveitis including glucocorticoids (*n* = 12, 48%), non-steroidal anti-inflammatory drugs (*n* = 4, 16%) and antiherpetic gels/ointments (*n* = 6, 24%). Additionally, 7 of the 25 cases (28%) were on systemic anti-inflammatory or antiherpetic treatment for their uveitis. This included valganciclovir (*n* = 3, with 1 also on prednisolone), valaciclovir (*n* = 2), tocilizumab and methotrexate (*n* = 1) and mycophenolate sodium (*n* = 1).

### Postoperative data

Early postoperatively (mean 2.8 ± 1.2 days), mean IOP reduced to 7.7 ± 3.0 mmHg (range 1.8 to 13 mmHg), representing a 77.7% ± 12.3% reduction (range 48.5 to 93%). IOP-lowering medications were discontinued in all eyes.

The mean postoperative follow-up amounted to 17.7 ± 11.3 months (range 3–44 months).

Fourteen of 25 eyes (56%) had an early postoperative IOP < 6 mmHg, but only 3 eyes (12%) for more than 2 weeks. Contact lenses were used for up to 3 weeks in three cases of hypotony with hyperfiltration. Transient (less than two weeks) hypotony-related maculopathy occurred in 5 eyes (20%) and choroidal effusions in 3 eyes (12%).

No cases of persisting hypotony complications were seen, including in eyes with prior cyclodestruction or aqueous shunts, nor in the eye with final IOP of 5 mmHg.

**Fig. 1:**
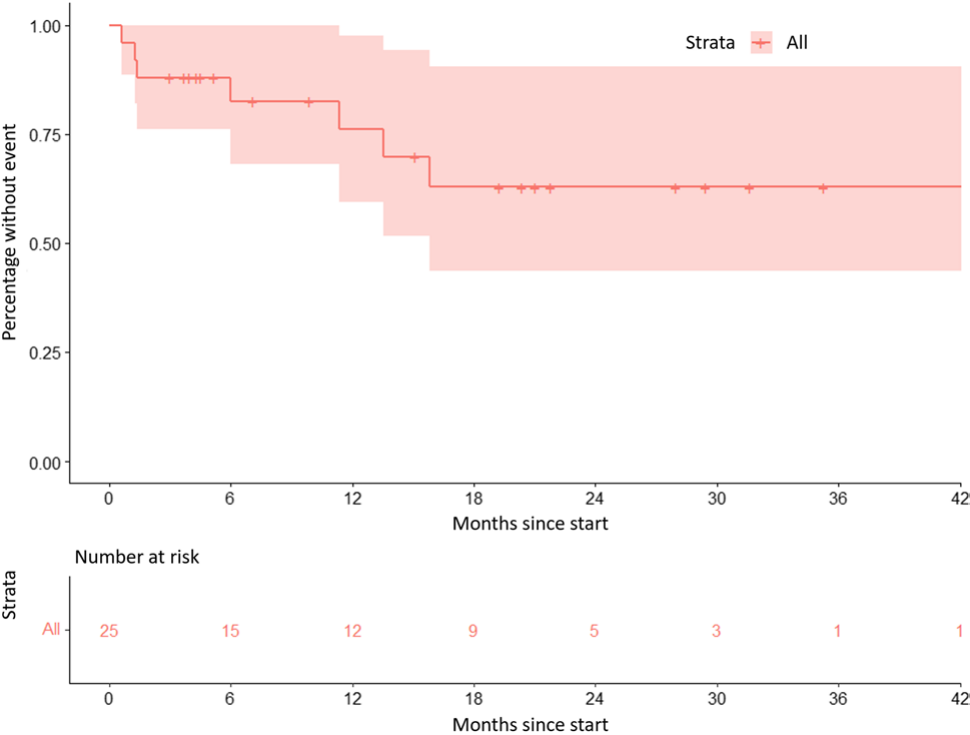
Kaplan–Meier curve showing the percentage of either open conjunctival bleb revision or restart of topical IOP lowering medication over time. Steps indicate events, ticks indicate eyes lost to follow-up. The shaded area represents the 95% confidence interval

**Fig. 2:**
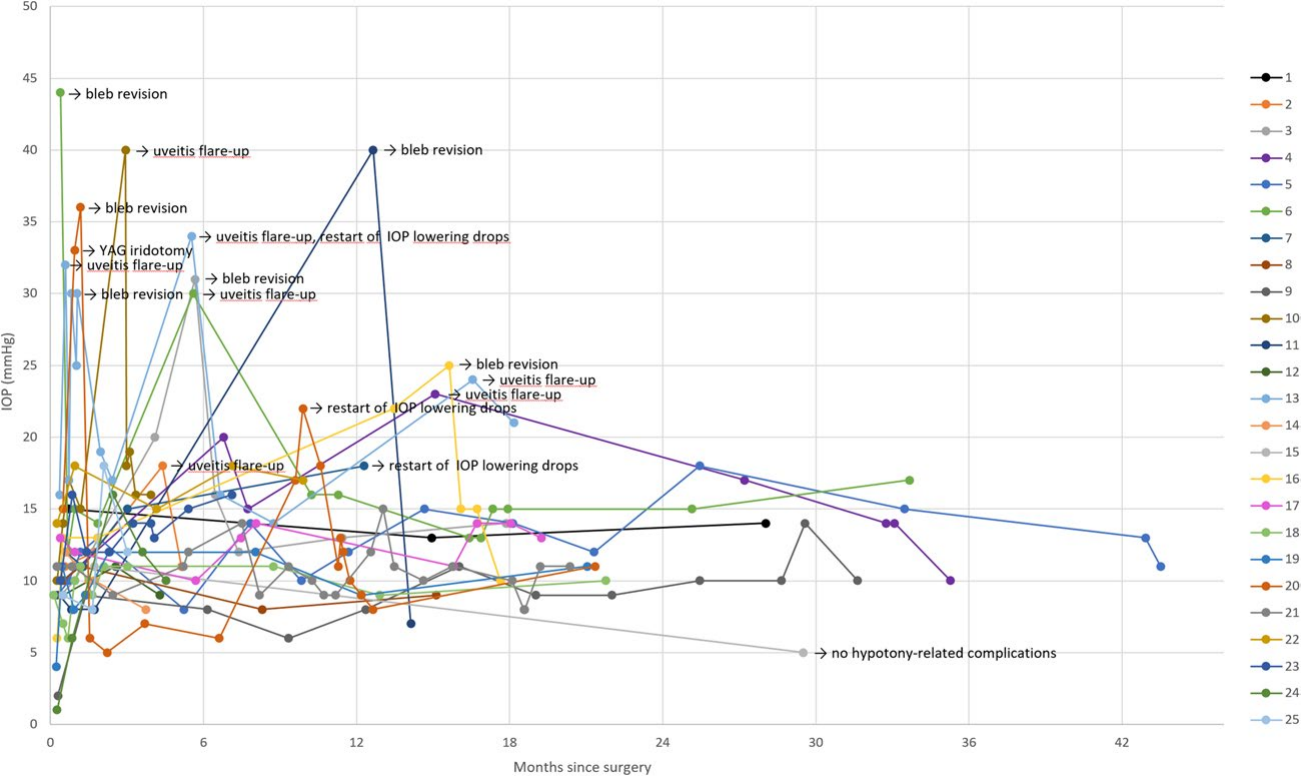
Course of postoperative IOP for every eye

Six eyes (24%) underwent open conjunctival bleb revision at a mean of 6.5 months (range 0.6 to 16.1 months) for impaired drainage due to occlusion of the stent lumen with Tenon’s fascia or subconjunctival scarring in 5 eyes and iris contact of the XEN^®^ stent in 1 eye (Figs. 1, and 2, Table 2). These eyes were considered failures.

In the patient who had iris contact with the XEN^®^ stent, we performed YAG iridotomy on postoperative day 29 after noting iris touch of the stent. However, the XEN^®^ implant function remained reduced afterwards. Therefore, on postoperative day 39, the patient ultimately underwent open conjunctival bleb revision surgery to reposition the stent.

Primary needling was performed in one patient, who thereafter had good bleb function and did not need open conjunctival bleb revision. Another patient underwent secondary needling one week after XEN^®^ implantation, but still needed open conjunctival bleb revision two and a half weeks after XEN^®^ implantation.

No eyes required further glaucoma surgery during the study period (complete failure).

Three eyes (12%) restarted topical antiglaucomatous medications at mean 8.9 months (range 5.5 to 11.4 months) (Fig. 2). Two had prior bleb revision; one remained a failure despite revision and medications. At final follow-up, the mean number of topical antiglaucomatous agents per eye was 0.2 ± 0.6 (93.1% reduction).

One of the post-Baerveldt eyes did require bleb revision and later restarted medications, indicating a more guarded prognosis after multiple surgeries. However, the other eye with a Baerveldt implant maintained an IOP of 11 mmHg at 44 months post-XEN^®^ implantation without further intervention.

At final follow-up (mean 17.7 months, 25 eyes), mean IOP was 12.0 ± 3.8 mmHg (range 5 to 21 mmHg), representing a 62.5 ± 18.9% reduction (range -5 to 84%). Follow-up data at approximately 6 and 12 months were available for 15 and 16 eyes, respectively (Table 1). At around 6 months, mean IOP was 13.5 ± 5.9 mmHg (range 6–30 mmHg) with a 57.8 ± 19.3% mean reduction. At approximately 12 months, mean IOP was 15.6 ± 8.3 mmHg (range 8–40 mmHg), with a 49.3 ± 28.1% mean reduction.

The IOP values at 6 and 12 months include eyes that subsequently underwent bleb revision or had IOP spikes from uveitis flare-ups.

At final follow-up, eyes that underwent bleb revision surgery had a mean IOP of 13.3 ± 5.1 mmHg (range 7–21 mmHg), representing a 54.4 ± 30.9% reduction (range -5 to 82%).

The course of postoperative IOP for each eye is presented in Fig. 2, IOP outcomes are summerized in Table 3.

Postoperatively, 9 of 25 eyes (36%) received intensified topical steroid treatment with up to hourly application for conjunctival injection or high uveitis flare-up risk. Three eyes (12%) received adjunctive atropine eye drops once or twice daily for transient hypotony with flattened anterior chamber.

One eye (4%) with recurrent herpetic uveitis and a history of multiple surgeries including Baerveldt implant developed an early postoperative uveitis flare-up with IOP increase to 34 mmHg and macular edema, requiring intensified topical, oral and intravitreal steroids as well as oral valaciclovir. This eye also showed early bleb dysfunction and underwent surgical revision at 1.4 months. The cause of bleb dysfunction in this case was unclear, but was unlikely due to luminal occlusion by inflammatory cells and debris, since there was free flow through the stent during revision surgery. The same patient restarted long-term topical antiglaucomatous treatment 5.6 months after XEN^®^ implantation but failed a final IOP ≤ 18 mmHg.

Three other eyes had temporary IOP spikes > 18 mmHg (less than 2 consecutive visits) during uveitis flare-ups despite functioning blebs but settled with anti-inflammatory/antiherpetic treatment.

One eye developed cystoid macular edema responsive to topical steroids.

Mean decimal visual acuity was 0.6 ± 0.4 preoperatively, 0.6±0.3 at the 1-year follow-up and 0.6 ± 0.3 at the last follow-up, respectively (Table 1).

At final follow-up, 18 eyes (72%, 95% confidence interval (CI): 50–88%) achieved complete success (IOP ≤ 18 mmHg without medications), one eye (4%, 95% CI: 0–20%) qualified success and six eyes (24%, 95% CI: 9–45%) failed (Table 4).

**Table 4:** Cumulative success rates at different times after surgery

	around 6 months after XEN^®^ implantation			around 12 months after XEN^®^ implantation			at final follow-up (after 17.7 ± 11.3 months)		
Complete success	11/15 eyes	73.3%	95% CI: 45–92%	10/16 eyes	62.5%	95% CI: 35–85%	18/25 eyes	72.0%	95% CI: 50–88%
Qualified success	0/15 eyes	0.0%	95% CI: 0–22%	1/16 eyes	6.25%	95% CI: 0–30%	1/25 eyes	4.0%	95% CI: 0–20%
Failure	4/15 eyes	26.7%	95% CI: 8–55%	5/16 eyes	31.25%	95% CI: 11–59%	6/25 eyes	24.0%	95% CI: 9–45%
Complete failure	0/15 eyes	0.0%	95% CI: 0–22%	0/16 eyes	0.0%	95% CI: 0–21%	0/25 eyes	0.0%	95% CI: 0–14%

Follow-up data for about 6 months and 12 months after XEN^®^ implantation are available for 15 and 16 eyes, respectively. At approximately 6 months, 11 of 15 eyes (73.3%, CI 95% 45–92%) had complete success and 4 eyes (26.7%, 95% CI 8–55%) failed. At around 12 months, 10 of 16 eyes (62.5%, 95% CI 35–85%) showed complete success, one eye (6.25%, 95% CI 0–30%) qualified success and five eyes (31.25%, 95% CI 0–21%) failed.

## Discussion

In this retrospective case series, XEN^®^-45 gel stent implantation effectively reduced IOP in eyes with medically uncontrolled uveitic glaucoma, including many with previous other glaucoma surgery. Preoperative IOP-lowering medications were reduced in all eyes and no secondary glaucoma surgery was required in the study period. However, XEN^®^-45 stent did not fully prevent IOP spikes during uveitis flares. The new XEN^®^-63 with a larger lumen may better control IOP during inflammation in future.

In this study, 24% of eyes required bleb revision surgery after XEN^®^ implantation. This highlights the need for close monitoring and management postoperatively.

Outcomes of bleb revision were generally favorable, with eyes achieving a mean IOP reduction of 54.4% after revision. This indicates bleb dysfunction can often be effectively treated with surgical revision.

However, one eye did fail to achieve IOP control even after bleb revision and restarting medications. This demonstrates revision may not always rescue a failing bleb or stent.

The efficacy of bleb revision surgery underscores the importance of early identification and prompt intervention when signs of dysfunction occur. Timely revision appears to offer a second chance at success.

There is one prospective case series of XEN^®^-45 implant for medically uncontrolled uveitic glaucoma (n = 24) with a 12 month follow-up by Sng et al. [8]. Compared to our study, needling was performed more often in this study. We sometimes perform needling in case of a subconjunctivally encapsulated XEN^®^ stent, but generally prefer open conjunctival bleb revision over needling, which explains the lower rate of needling in our study. Furthermore, postoperative needling in the Sng et al. study was performed as a slit-lamp procedure whereas we perform postoperative needling in the operating room. The rate of open conjunctival bleb revision was similar in both studies (5 of 24 compared to 6 of 25 eyes). However, it may be underestimatid in our study due to missing follow-up data. Sng et al. reported a low incidence of potencially sight-threatening complications, including bleb-related ocular infection and persistent hypotony.

Another retrospective case series (*n* = 37) showed effectiveness of the XEN^®^-45 stent also in urgent management of uveitic glaucoma without significant uveitis flare-up [9]. During a mean follow-up time of 16.7 months (range: 12–32 months) 13.5% of the patients underwent secondary glaucoma-related surgery, wich was not performed in any of our cases during a mean follow-up time of 17.7 months.

In patients with uveitic glaucoma, minimally-invasive iridocorneal angle based glaucoma surgery with the Trabectome^®^ was effective and feasible in a previous retrospective case series from our department (*n* = 24)[10], as well as case series from other departments [11, 12]. However, some of these patients over time need additional surgery. In this case series of XEN^®^-45 implantation, a majority of 72% (18/25) previously underwent Trabectome^®^ surgery. Kiessling et al. have shown that trabeculectomy ab interno with the Trabectome^®^ does not interfere with following XEN^®^-45 implantation in non-uveitic pseudophakic eyes [13]. Our case series is too small to prove a difference between eyes with or without previous Trabectome^®^ surgery. However, also in eyes with uveitic glaucoma, Trabectome^®^ does not seem to interfere with XEN^®^-45 implantation.

XEN^®^-45 implantation provides an additional outflow pathway to the subconjunctival space via the stent, whereas Trabectome^®^ surgery only reduces outflow resistance by removing trabecular meshwork in front of Schlemm’s canal. Therefore, XEN®-45 implantation can achieve a more pronounced reduction of IOP on the one hand, but has an increased risk of postoperative hypotony compared to Trabectome^®^ surgery on the other hand. Consequently, XEN^®^ implantation should be compared to classical trabeculectomy rather than Trabectome^®^ surgery.

Results of classical trabeculectomy ab externo in uveitic glaucoma show some variability: Some studies found good long-term outcomes comparable to nonuveitic glaucoma [14, 15], even without antimetabolite use [16]. However, in another study, trabeculectomy with mitomycin C in uveitic glaucoma patients was not as successful as in nonuveitic glaucoma patients [17]. Eyes of uveitis patients probably are more prone to tissue fibrosis and scarring, increasing their risk for outflow obstruction after trabeculectomy ab externo.

Compared to classical filtration surgery, XEN^®^ implantation is less invasive with reduced trauma to the iris, sclera and conjunctiva, potentially resulting in less postoperative inflammation [18–21]. Sutures are not necessary except for bleb revision with conjunctival dissection. The duration of surgery is usually shorter with the XEN^®^ implant. Both procedures show comparable efficacy [22, 23], leading to significant reduction of IOP and IOP-lowering medication burden. Although short-term IOP reduction is generally more pronounced after XEN^®^ implantation, long-term IOP reduction is more pronounced after trabeculectomy [18, 19]. Marcos Parra et al. showed that complications are more frequent after trabeculectomy for primary open-angle glaucoma compared to XEN^®^ implantation [24]. However, other studies showed that reintervention rate, including both needling and revision surgery with conjunctival dissection, is higher after XEN^®^ implantation compared to other glaucoma filtering surgery [25, 26]. Basílio et al. showed non-inferiority of medium-term quality of life after XEN^®^ implantation compared to trabeculectomy after median 12 months [27].

The introduction of foreign material into the eye may be seen as a disadvantage, but the organic material of the XEN^®^ gel stent appears to be well tolerated even in uveitic eyes. The small 45 µm inner diameter of the implant suggests a potential risk of occlusion by inflammatory material like fibrin or clogging by inflammatory cells and debris. However, this study could not demonstrate evidence of luminal occlusion or clogging by inflammation.

The Preserflo^®^ is another stent for drainage of intraocular fluid to the subconjunctival space. It differs from XEN^®^ in terms of material (biocompatible, synthetic polymer), lumen diameter (70 µm) and route of implantation (ab externo). A retrospective case series by Triolo et al. showed favorable efficacy and safety of Preserflo^®^ in the treatment of refractory uveitic glaucoma after three years [28].

Glaucoma drainage device implantation is more invasive compared to XEN^®^ implantation, but has been shown to be effective and safe for uveitic glaucoma [29–31]. In a retrospective analysis, our study group analyzed the success of Baerveldt 250 implantation in uveitic glaucoma when other interventions had already failed [32]. No further glaucoma-related surgery was required in 75% of eyes within a follow-up period of almost 2 years and at final follow-up 58.3% maintained or improved their visual acuity. In a retrospective study comparing Ahmed versus Baerveldt glaucoma drainage devices for uveitic glaucoma, a higher complete success rate and significantly greater reduction in mean IOP and number of medications were observed with the Baerveldt, but with a higher rate of complications including hypotony [33]. These drainage devices remain as an option after XEN^®^ failure or if XEN®^®^ implantation is not deemed to succeed, for example in cases of pre-existing subconjunctival scarring.

Another option of glaucoma surgery in cases of subconjunctival scarring are supraciliary implants. However, there is insufficient data on the use of supraciliary shunts in uveitic glaucoma patients. Additionally, there are several reports of granulomatous inflammation or foreign body reaction after CyPass^®^ implantation, documented histologically after stent explantation, often due to endothelial cell loss [34, 35].

Our study is limited by the small number of cases and short follow-up period. Some patients were followed outside the study center during the follow-up period, leading to incomplete data capture, which may under- or overestimate failure rates and IOP values at later follow-ups. However, it is reassuring that IOP values remained between 5–21 mmHg among patients with available data after 18 months. No additional revision surgeries were reported beyond 16 months, suggesting stable long-term IOP control was achieved for some patients. Furthermore, the evaluation of surgical success is limited to IOP values and does not provide data on stabilization or progression of glaucomatous damage. There was broad variation in baseline parameters including glaucoma severity, uveitis type, prior interventions, and lens status. Multiple prior glaucoma surgeries, as was the case in most of our cohort, is a prognostically unfavorable factor [36].

It should be noted that the XEN^®^ implant is not yet approved for uveitis patients (off-label use). However, our study provides further evidence for the efficacy of this approach.

## Conclusion

In conclusion, this retrospective case series provides real-world data on outcomes of the XEN^®^-45 gel stent for medically uncontrolled uveitic glaucoma. The study found XEN^®^ implantation effectively reduced IOP from a mean of 35.3 mmHg preoperatively to 12.0 mmHg at final follow-up of 17.7 months on average. This represented a mean IOP reduction of 62.5% in the full cohort. Complete success with IOP ≤ 18 mmHg without medications was achieved in 72% of eyes.

Bleb revision surgery was required in 24% of eyes. It generally had good outcomes, with eyes that underwent revision achieving a mean 54.4% IOP reduction and mean IOP of 13.3 mmHg at final follow-up.

The XEN^®^-45 stent may not sufficiently control IOP to completely avoid spikes during uveitis flare-ups, as 12% of eyes experienced transient IOP elevations despite patent blebs. The newer XEN^®^-63 stent, with a larger lumen diameter, may help better control IOP during inflammation.

In eyes with uveitic glaucoma, the XEN^®^ stent shows promise as a minimally invasive option for IOP reduction but requires meticulous postoperative management. Further research with extended follow-up is warranted to elucidate long-term surgical success, necessity of postoperative interventions and complications for this complex patient population.
